# Dimensional structure of the items from The Swedish Demand-Control-Support Questionnaire (DCSQ) used in The HUNT Study

**DOI:** 10.1371/journal.pone.0308611

**Published:** 2024-09-26

**Authors:** Jonil Tau Sperstad, Grahame Coleman, Karianne Muri, Eystein Skjerve, Kjersti Selstad Utaaker, Magnhild Oust Torske

**Affiliations:** 1 Faculty of Biosciences and Aquaculture, Nord University, Bodø, Norway; 2 Faculty of Biosciences and Aquaculture, Nord University, Steinkjer, Norway; 3 Faculty of Science, The University of Melbourne, Melbourne, Australia; 4 Faculty of Veterinary Medicine, Norwegian University of Life Sciences, Ås, Norway; Guizhou Medical University, CHINA

## Abstract

**Objective:**

The Swedish Demand-Control-Support Questionnaire (DCSQ) is used to measure psychosocial work environment. Nine of the original 17 DCSQ items were used in the Trøndelag Health Study (HUNT) in 2017–2019 (HUNT4); three items from each of the three dimensions of *demand*, *control*, and *support*. The goal of this paper was to assess the internal reliability and dimensional structure of the nine DCSQ items used in HUNT4.

**Method:**

HUNT4 participants registered with an occupation, and who had responded to all DCSQ items were included in the sample. Internal reliability and consistency of the nine DCSQ items were tested using composite reliability and item total correlation. A confirmatory factor analysis (CFA) was performed to determine whether the nine DCSQ items used in HUNT4 had a similar factor structure as the original 17 items. CFA was performed on the sample as a whole, before testing the models on different occupational groups to check if the dimensional structure remained the same.

**Results:**

Of 56,041 HUNT4 participants (participation rate: 54%), 21,187 had a known occupation and responded to all nine DCSQ items. The mean age of the sample was 50.6 years (SD = 10.2), and the sample consisted of 57.3% females and 42.7% males. The best model fit was achieved by removing the item “Work creativity” (λ = 0.398, item total correlation 0.334) from the model. The same three-factorial structure as in the original DCSQ was seen with the remaining eight DCSQ items, with good internal consistency of all three dimensions (composite reliability ranged from 0.709 to 0.851). This dimensional structure remained the same when tested on all occupational groups.

**Conclusion:**

The results indicate that the shortened version of the Swedish DCSQ used in HUNT4 can be used to assess aspects of *demand*, *control*, and *social support* at work.

## Introduction

Occupational stress refers to the physical, mental and emotional strain experienced by individuals as a result of the demands they encounter in their work environment [1]. The effects of a stressful work environment have been extensively explored over the past decades, and several models have been created to explain occupational stress. One of the most widely used models is the Job Strain model [2], also known as the Job Demand-Control (JDC) model, first developed and presented by Robert Karasek in 1979 [3]. Demand refers to the psychological stressors associated with workload, whilst control is a measure of the worker’s autonomy over work tasks [3]. According to Karasek’s model, workers experiencing high psychological demands and low control at work have high strain jobs and are more likely to experience poor physical and mental health [3,4]. The model was further developed in the 1980’s to include social support as a third dimension, resulting in the Job Demand-Control-Support model (JDCS) [5]. Support refers to how pleasant the work environment is and how well a worker gets on with and is supported by fellow workers and supervisors [6]. The JDCS model theorises that high social support can act as a buffer, compensating for some of the negative consequences of high work demands and/or low control. Research has found equal support for both the JDC and JDCS models [2] and confirmed an additive effect of the three dimensions on general psychological well-being [2,7]. The combination of high work demand, low work control and low social support is associated with the greatest risk of poor physical and psychological health [5].

The Swedish Demand-Control-Support Questionnaire (DCSQ) is a shortened version of the questionnaire based on the JDCS model. It is a self-administered questionnaire consisting of 17 items measuring social and psychological characteristics of work [8–10]. The 17 items are grouped into the same three dimensions defined in the JDCS model: *demand*, *control*, and *support*. The dimensional structure has been tested on several translations of the questionnaire [6,11–14]. Mauss et al. evaluated the dimensional structure of an English and a German version of the questionnaire, and exploratory factor analysis (EFA) showed that the 17 items loaded on the same three factors as described in Theorell’s model [13]. The same three-factorial structure was found in a Spanish version of the questionnaire [14].

The *control* dimension is also referred to as the *decision latitude* dimension, and the items in this dimension represent two underlying aspects of control at work; *decision authority* and *skill discretion* [4]. Decision authority refers to the worker’s ability to make decisions, and skill discretion refers to the worker’s ability to utilise and develop skills [8]. Some studies have shown that *decision authority* and *skill discretion* form two distinct dimensions, implying that a four-factor structure might be more appropriate than the three-factor structure [11,12]. Sanne et al. demonstrated that the Norwegian translation of the Swedish DCSQ has a three-factor solution for an occupationally heterogenous sample, but they did find evidence of a four factor-solution when looking at certain occupational groups [6].

The JDC and JDCS models have inspired and influenced the creation of other models, such as the Job Demand-Resource (JDR) model [15]. Despite the emergence of other models, the JDCS model remains one of the central models in this field of research. The simplicity and robustness of the model have been pointed out as factors allowing for the model to easily be used and incorporated as part of larger questionnaires [16]. The items can be of particular value in health-based surveys, as the model is so well established in the research of associations between occupational stress and various health outcomes [5,10,17].

The Norwegian version of the DCSQ has been used in different population-based health studies such as the Hordaland Health Study [6] and the Trøndelag Health study (the HUNT Study) [18]. Nine of the 17 items in the Swedish DCSQ were included in the last two surveys of the HUNT Study (HUNT3 [2006–2008] and HUNT4 [2017–2019]) [19]. To the authors’ knowledge, there is only one scientific publication based on the HUNT data that uses the nine DCSQ items [20], and the dimensional validity of these nine items has not been evaluated. The HUNT Study is a comprehensive cohort study with more than 230,000 individuals participating since 1984 [18] and could serve as a valuable data source for research on psychosocial work environment. However, interpretation of results presupposes that the factor structure of the nine-item version of the DCSQ is established. Establishing the dimensional structure of the DCSQ items used in HUNT can facilitate future use of these items from the HUNT study. Furthermore, to keep the burden of respondents in surveys down, using a shorter version of the DCSQ may be of interest to other researchers. Therefore, the aim of this study is to assess the dimensional structure and the internal consistency of the nine DCSQ items used in The HUNT4 Survey.

## Materials and methods

### Study population

All registered residents of Nord-Trøndelag County aged 20 and above were invited to participate in The HUNT4 Survey (2017–2019). Invitations were sent out by mail together with the first questionnaire (Q1). Q1 could be completed online (by using a personal username and identification number (PIN) provided by HUNT to log on) or in printed form. Participants were invited to attend a health screening at a mobile field station, where completed printed versions of Q1 were returned. At the field station participants were interviewed and given a second baseline questionnaire (Q2). This questionnaire contained gender and age-specific questions regarding health, work and lifestyle, and participants were, therefore, given different versions depending on their age and sex. Only those in the age group 30–69 were given a Q2 version containing the nine DCSQ items. Participants were asked to respond to the DCSQ items if they were currently working or had previously worked. Those who had previously worked together with others, or were currently working together with others, were asked to respond to the *support* items. More details about the HUNT Study and information on the data collection process done by HUNT is available elsewhere [18,21,22].

At the field station, participants were asked what their current main occupation was, or had been in the past if they were not currently occupationally active. The occupations were classified according to the Norwegian Standard Classification of Occupations (STYRK 98), which is based on the International Standard Classification of Occupations (ISCO-88) [23].

**Fig 1:**
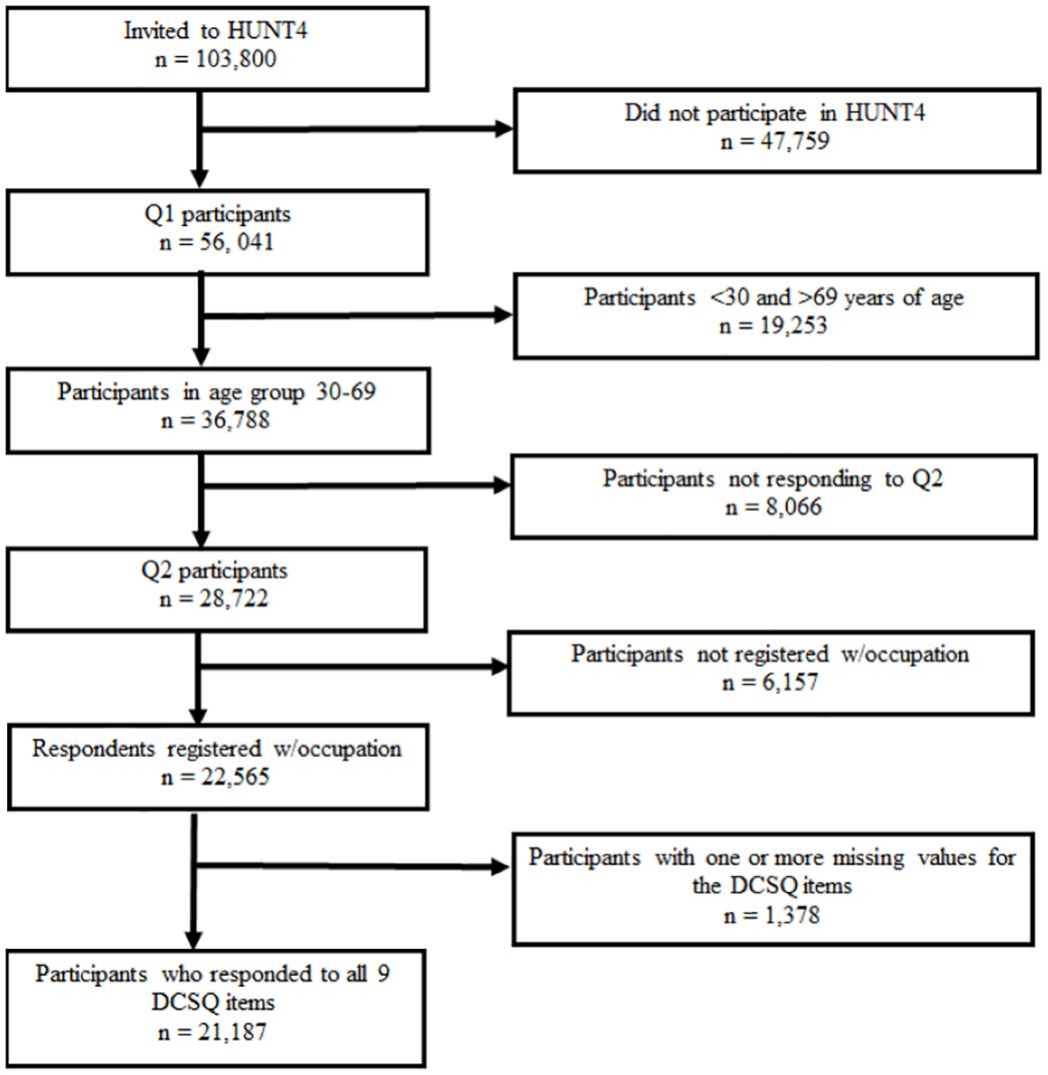
Flow-chart of the study sample selection process. HUNT4 = The Trøndelag Health Study in 2017–2019. Q1 = Questionnaire 1. Q2 = Questionnaire 2. DCSQ = the Demand-Control-Support Questionnaire.

The inclusion criteria of the current study were HUNT4 participants who: 1) were 30–69 years old, 2) were registered with an occupation, and 3) had completed all the DCSQ items. The selection of study participants is shown in Fig 1. Of the 103,800 residents of Nord-Trøndelag County who were invited to HUNT4, 56,041 individuals responded to Q1 (response rate 54%). Of these, 36,788 were between 30–69 years old and received a version of Q2 that included the DCSQ items. Participants not registered with an occupation (n = 6,157), or with one or more missing responses to the DCSQ items (n = 1,378) were excluded. This left 21,187 individuals in the study sample.

### The Demand-Control-Support Questionnaire

Table 1 provides an overview of the nine DCSQ items used in HUNT4, and the eight DCSQ items that were not used in HUNT4, with the underlying latent variables explaining the inter-item correlations as described by Theorell and others [6,10,13]. Of the nine DCSQ items used in HUNT4, there are three items representing each of the three factors. If the *control* factor is split into *skill discretion* and *decision authority*, they are represented by one and two items respectively. The selected items used in HUNT4 makes the demand dimension workload-oriented, and the support items focus only on the support of co-workers and not supervisors. The items are scored on Likert scales identical to the original questionnaire. The items of the *demand* and *control* factors are scored on a frequency-based Likert scale ranging from 1 (yes, often) to 4 (no, almost never). The items of the *support* dimension are scored on an agreement-scale, ranging from 1 (strongly agree) to 4 (strongly disagree).

**Table 1:** Description of the items from the Swedish Demand-Control-Support Questionnaire (DCSQ).

Factor/Latent variable		DCSQ item	Shortened name of DCSQ item
**Demand**		*Does your job require you to work very fast*?	*Work fast*
		*Does your job require you to work very hard*?	*Work hard*
		*Does your job require too great a work effort*?	*Work effort*
		Do you have sufficient time for all your work tasks?	
		Do conflicting demands often occur in your work?	
**Control**	**Skill discretion**	*Does your job require creativity*?	*Work creativity*
		Does your job require skills?	
		Does your job require doing the same tasks over and over again?	
		Do you have the opportunity to learn new things in your work?	
	**Decision authority**	*Do you have the possibility to decide for yourself how to carry out your work*?	*Work how*
		*Do you have the possibility to decide for yourself what should be done in your work*?	*Work what*
**Support**		*There is good collegiality at work*	*Work collegiality*
		*My co-workers are there for me (support me)*	*Work support*
		*I get along well with my co-workers*	*Work welfare*
		There is a quiet and pleasant atmosphere at my place of work	
		People at work understand that I may have a “bad” day	
		I get along well with my supervisors	

The nine DCSQ items in the grey coloured boxes and written in italics are the items used in the Trøndelag Health Study in 2017–2019 (HUNT4). The remaining eight DCSQ items (white background) were not included in the HUNT4 survey. The shortened names are abbreviations used to refer to the DCSQ items in the text.

### Statistical analysis

The statistical analyses were performed in R version 4.2.2 [24]. The packages *lavaan* [25], *semTools* [26], *multilevel* [27] and *psych* [28] were used for the factor analyses and internal reliability testing. Graphics were created using *lavaanPlot* [29]. The missing values were explored using the Little’s missing completely at random (MCAR) test [30] from the *naniar* package [31].

#### Internal consistency

Internal consistency was evaluated through composite reliability and item-total correlation. Values above 0.7 for composite reliability indicate good internal consistency [32]. The 95% confidence interval (CI) was estimated by bootstrapping with 1000 replications. Item-total correlation values above 0.3 are indicative of good internal consistency [33].

#### Construct validity

Exploratory factor analysis (EFA) using principal component analysis with oblique rotation was initially performed to examine the underlying factor structure of the nine DCSQ items. Factors with an eigenvalue >1 were retained, and factor loadings >0.45 were considered satisfactory [34].

To verify that the items had a similar factor structure as the original DCSQ, confirmatory factor analysis (CFA) was carried out. A weighted least square mean and variance (WLSMV) estimator was used, as this is suitable for ordinal data [35,36].

Model 1 was created by setting the items to load on the same three factors that they load on in the original 17 items questionnaire: *demand*, *control* and *support*. Covariance between factors was permitted. The factor loading and the residual variance were assessed for each item to evaluate its role in the model. Several fit indices were used to assess the model’s goodness of fit. The comparative fit index (CFI) and the Tucker-Lewis index (TLI) were used. Values >0.95 for CFI and TLI indicate a good fit [37]. In addition, the root mean square error of approximation (RMSEA) was used to assess model fit. RMSEA values <0.05 indicate a close fit, and values >0.08 indicate a poor fit [37]. Lastly, the standardised root mean squared residual (SRMR) was used to assess the size of the residuals where values <0.05 are considered good, and values between 0.05 and 0.08 are considered acceptable [37].

Adjustments were made to Model 1based on the fit indices and the model parameters to create a model with improved goodness of fit. The item with the lowest factor loading and highest residual variance (“Work creativity”) was removed to create Model 2. The factor loading and residual variance was assessed for the remaining items in Model 2, and the same fit indices (CFI, TLI, RMSEA and SRMR) were used to assess the goodness of fit of the model. A chi-square difference test was performed to compare the fit between the two models.

CFA was initially performed on the sample as a whole, before testing Model 1 and Model 2 on each of the ten main occupational groups (Table 2) to check whether the dimensional structure remained the same in the different occupational groups.

**Table 2:** Characteristics of the study sample. The Trøndelag Health Study 2017–2019 (HUNT4).

		n	%
**Total sample**		21,187	100
**Sex**	Women	12,146	57.3
	Men	9,041	42.7
**Highest level of education completed** ^a^	Primary and lower secondary school	844	4.0
	Secondary school^b^	9,887	46.7
	College or university	10,415	49.3
**Main occupational group (ISCO groups)**	ISCO 0 –Armed forces and unspecified	221	1.0
	ISCO 1 –Legislators, senior officials and managers	1,795	8.5
	ISCO 2 –Professionals	3,160	14.9
	ISCO 3 –Technicians and associate professionals	5,725	27.0
	ISCO 4 –Clerks	1,038	4.9
	ISCO 5 –Service workers and shop/market sales workers	4,435	20.9
	ISCO 6 –Skilled agricultural and fishery workers	982	4.6
	ISCO 7 –Craft and related trades workers	1,820	8.6
	ISCO 8 –Plant and machine operators and assemblers	1,288	6.1
	ISCO 9 –Elementary occupations	723	3.4

ISCO = International Standard Classification of Occupations.

^a^Not all individuals in the final study sample had registered information on education (missing: n = 41 (2%)). The reported percentages are based on study participants registered with an educational status (n = 21,146).

^b^Includes Academic/vocational school 1–3 years and vocational school/apprentice 3–4 years.

### Ethical considerations and data handling

The HUNT4 survey was considered by the Regional Committee for Medical Research Ethics Central Norway (REK Central) to be a health registry, and not a health research project. Consequently, the data collection did not require REC approval, according to the Act on Medical and Health Research [38]. The project the present study is a part of has been evaluated by the Regional Committee for Medical Research Ethics Northern Norway (REK North) twice, in 2019 (reference number 34574) and 2021 (reference number 256719). REK North considered the project not to be medical research as defined by the Act on medical and health research [39], thus not requiring REK approval. The project has been assessed by the Norwegian Centre for Research Data (NSD, now Norwegian Agency for Shared Services in Education and Research (SIKT)) in 2020 (reference number 923148) and has a Data Protection Impact Assessment (DPIA). To protect the confidentiality of the HUNT4 participants the data were pseudonymised by giving all participants a project specific identification number (PID) before it was sent to the researchers. The data were made accessible to the researchers on the 8^th^ of March 2022.

## Results

### Description of study sample

Of the 21,187 participants, the mean age was 50.6 years (standard deviation: 10.2, median: 51.4) and 57.3% were females. A higher percentage of the females in the study population had completed a university degree compared to the male population (females: 31.5%, males: 17.7%). Characteristics of the study population are summarised in Table 2.

### Missing data

Of the 22,565 participants registered with an occupation, 1,378 (6.1%) had one or more missing responses to the DCSQ items. There were approximately 4,3% missing responses for each of the support items, whilst all the other items had less than 3% missing. A significant MCAR test confirmed that the data were not missing completely at random (p = 0.01). Inspection of the missing values showed that there was a notably higher percentage of missing among the support items in occupational group 6, where 17.5% of the respondents had left one or more of the items unanswered. Listwise deletion of the 1,378 participants with one or more missing, was performed.

### Internal consistency and reliability

The composite reliability values indicated good internal consistency of all three factors, with values above 0.7 (Table 3). The item-total correlation confirmed good internal reliability of most items. However, the item “Work creativity” stood out with a value closer to the cut-off point of 0.3 than the other items. Removing this item from the control dimension changed the correlation between the two remaining items, “Work how” and “Work what”, to 0.670, and increased the composite reliability to 0.839 (95% CI: 0.834–0.844).

**Table 3:** Internal reliability and consistency of the Swedish Demand-Control-Support items used in the Trøndelag Health Study in 2017–2019 (HUNT4).

		Composite reliability (95% CI)	Item-total correlation
**Demand**		0.709 (0.702–0.716)	
	Work fast		0.556
	Work hard		0.542
	Work effort		0.474
**Control**		0.737 (0.730–0.744)	
	Work creativity		0.334
	Work how		0.622
	Work what		0.601
**Support**		0.851 (0.846–0.856)	
	Work collegiality		0.713
	Work support		0.716
	Work welfare		0.741

Composite reliability with 95% confidence interval (CI), and item-total correlation.

### Exploratory factor analysis

Exploratory factor analysis indicated a three-factor solution, with the same factorial structure of the items as in the original DCSQ. The item “Work creativity” had a factor loading of 0.448, while all other items had loadings between 0.648 and 0.946 (S1 Table).

### Confirmatory factor analysis

#### Model 1

The results from testing Model 1 on the whole sample are summarized in Table 4. Most items had high factor loadings. The item “Work creativity” stood out from the rest with the lowest factor loading (λ = 0.398) and the highest residual variance (δ = 0.841). All three factors showed significant covariation (*p*≤0.015); representing a negative relationship between *demand* and *support*, and *demand* and *control*, and a positive relationship between *support* and *control*.

**Table 4:** Confirmatory factor analysis of the Swedish Demand-Control-Support Questionnaire items used in the Trøndelag Health Study in 2017–2019 (HUNT4).

	Model 1		Model 2	
	λ	δ	λ	δ
**Demand**				
**Work fast**	0.794	0.369	0.806	0.351
**Work hard**	0.762	0.420	0.753	0.432
**Work effort**	0.630	0.604	0.626	0.608
**Control**				
**Work creativity**	0.401	0.839	-	-
**Work how**	0.919	0.155	0.946	0.106
**Work what**	0.847	0.283	0.821	0.326
**Support**				
**Work collegiality**	0.880	0.225	0.880	0.225
**Work support**	0.889	0.210	0.889	0.210
**Work welfare**	0.949	0.099	0.949	0.099
**Factor correlation**				
**Demand-Control**	-0.022		-0.105	
**Demand-Support**	-0.084		-0.084	
**Control-Support**	0.197		0.203	
**Goodness of fit indices**				
**TLI**	0.966		0.995	
**CFI**	0.978		0.997	
**RMSEA (90% CI)**	0.091 (0.089–0.094)		0.039 (0.036–0.041)	
**SRMR**	0.064		0.025	

Standardised factor loadings (λ), standardised factor correlations and standardised residual variance (δ), and goodness of fit indices. Model 1 included the item “Work creativity”. This item was removed from Model 2. TLI = Tucker Lewis index. CFI = Comparative fit index. RMSEA = Root mean square error of approximation. SRMR = Standardised root mean squared residual.

Both CFI (0.973) and TLI (0.959) indicated a good fit of the model. However, the RMSEA value of 0.103 (90% CI: 0.101–0.105) indicated a poor fit between the model created and the observed data. SRMR was acceptable (0.069).

#### Model 2

**Fig 2:**
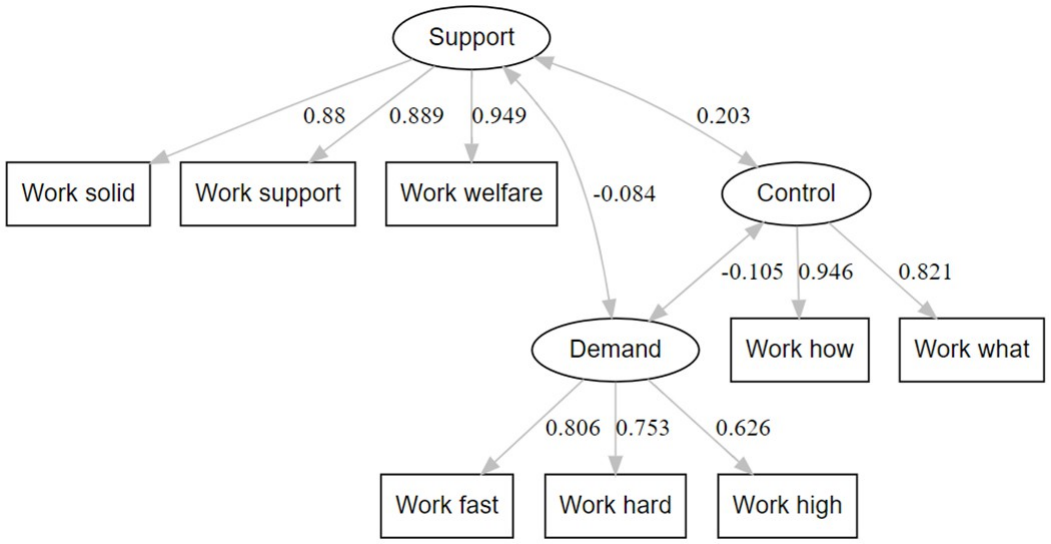
Graphical display of the three-factor solution with the factor loadings of each of the Demand-Control-Support Questionnaire items from the HUNT4 Survey (2017–2019), with the item "Work creativity" removed (Model 2).

Due to the low factor loading and high residual variance, a second model (Model 2) was created where the item “Work creativity” was removed to try and improve the model fit. The factor loading of the eight items retained in Model 2 remained high, and the residual variance remained low. All the fit indices improved in Model 2: TLI (0.995), CFI (0.997), RMSEA (0.039) and SRMR (0.025). The three factors showed the same significant covariance as seen in Model 1 (p<0.001) (Table 4). Model comparison showed a significant decrease in Chi-square between Model 1 (χ^2^ = 3245.9) and Model 2 (χ^2^ = 344.2) (p<0.001). A graphic display of model 2 with the factor loadings is shown in Fig 2.

#### Dimensional structure of the occupational groups

Testing the two models on the main ISCO occupational groups showed the same three-dimensional structure for all the ten groups. “Work creativity” remained the item with the lowest factor loading, with a value of <0.4 for most groups. Removing “Work creativity” from the model improved the model fit, and the factor loadings remained high for all occupational groups (S2 and S3 Tables).

## Discussion

The results indicate that the nine items used in HUNT4 appear to provide information about the same underlying aspects of psychosocial work environment as in the original DCSQ model. The HUNT Study is a valuable and commonly used data source for health research, however, research looking at psychosocial work environment using the nine DCSQ items has been very limited. Establishing the dimensional structure of these nine DCSQ items could simplify the interpretation and use of these variables in future research on psychosocial work environment. In a questionnaire-based health study, such as the HUNT Study, limiting the number of questions to ensure that the burden on the study participants is as low as possible is important [40]. This may explain why the full 17 items questionnaire was not used in the HUNT study. It is not known why these specific nine items were selected from the 17 original questions, but these items have the highest factor loadings in the study by Sanne et al. [6].

Our findings show that a three-dimensional structure without the item “Work creativity” creates a better model than when all nine DCSQ items are used. The eight remaining items had high factor loadings on the three dimensions they were hypothesised to load on. The low factor loading, and high residual variance of the item “Work Creativity” may indicate a four-factor solution. Several studies support the four-factor structure of the DCSQ model, arguing that the *control* factor should be split in two separate dimensions: skill discretion and decision authority [4,6,12]. This separates the item “Work creativity” from the other two *control* items, to form two different dimensions. “Work how” and “Work what” are measures of *decision authority*, whilst “Work creativity” belongs to *skill discretion*. Despite positively influencing job satisfaction and motivation [41], research show that aspects of skill discretion may be more of a measure of job demands than a measure of control and autonomy [42] and including these items in the job control dimension may make it challenging to interpret the results in terms of job control [43]. This could explain why “Work creativity” stands out with a considerably lower factor loading and higher residual variance than the other items in Model 1. Based on these results we cannot conclude if the item is a measure of job control. However, creativity in the workplace is associated with job satisfaction [44] and the item should still be considered a valuable source of information in future studies on work environment using The HUNT Study.

The correlation between *skill discretion* and *decision authority* has been shown to vary between occupational groups [45]. Sanne et al. found that a three-factor solution is suitable for an occupationally heterogenous sample, however, for a more homogenous group, a four-factor solution might be more appropriate [6]. Even though Sanne et al. also used the Norwegian translation of the DCSQ model, direct comparison with our study is challenging due to the choice of different statistical methods, and the different number of items, as they used the full version of the DCSQ. Though our results may indicate that the *control* items are better separated into two factors in both an occupationally heterogenous and homogenous sample, the reduced number of items limits the ability to explore this further as the number of items per factor would be too low, creating an under-identified model [46].

The items show good internal consistency, with the *support* dimension having the highest reliability, corresponding with other studies [11,13,14]. However, the internal reliability of the *control* dimension is slightly lower in the present study compared to other studies [6,13]. This value is probably affected by “Work creativity”, and removing the item from the *control* dimension increases internal reliability of the factor.

The nine questions used in HUNT4 are already a reduced version of the existing Swedish Demand-Control-Support Questionnaire, and further reducing the number of items could result in the loss of valuable data. Reducing the number of variables in the model to eight creates a better model fit, but the construct validity of the model might not be as good [47], as factor analysis tends to perform better when there are more variables representing each factor [46]. By using only some of the items from each dimension, the focus of the dimensions is altered. All three items in the support dimension are focused on support from co-workers only and do not take into account the importance of leaders and leadership in the perceived workplace support. Lack of support from supervisors at work has been linked to decreased well-being and health of workers [48]. However, other research suggest support from supervisors does not significantly impact psychological distress among workers [49]. Workload was highlighted by Karasek and Theorell to be the main component of job demands, and all three items in this shortened version of the DCSQ are workload-oriented [3]. However, it has been suggested that the focus on workload and time management in this dimension, may not fit well with all occupational groups, and that the model could benefit from more occupation-specific measurements [7]. Despite this, our results indicate that the shortened version of the DCSQ has a similar dimensional structure in all major ISCO groups.

The HUNT Study has several qualities that make it useful for epidemiological research, including its size, high participation rate, repeated measurements enabling comparisons over time, and the ability to merge data with other registries [18,22]. Establishing that the shortened version of the DCSQ used in HUNT4 has the same dimensional structure as the original 17-item version, will hopefully facilitate and encourage the use of these items in future studies on psychosocial work environment using data from the HUNT Study. It also supports the possibility of using an abbreviated version of the DCSQ in other data collections where questionnaire space is at a premium and where it is necessary to limit the number of questions the participants have to answer. This may limit the respondent burden and may improve response rates, improve reliability of answers and decrease the amount of missing data compared to very long and comprehensive questionnaires [40]. However, even though the dimensional structure of the nine items is consistent with the original DCSQ, more research is needed to investigate whether the factors in the reduced questionnaire correlate well with the factors in the full 17-items questionnaire. This would add valuable information about whether the reduced factors are representative of the original DCSQ or if the shortened version represents only parts of the original questionnaire. Establishing this would assist the interpretation of results from studies using the DCSQ items from the HUNT Study.

### Strengths and limitations

The large sample size is a strength in this study which increases the reliability of the results. A large sample size is preferred for factor analysis, especially when there are relatively few items per factor [34]. The broad inclusion criteria of HUNT4 allowed for a heterogenous study population representing people of different educational backgrounds and all main occupational groups, as well as a relatively well-balanced gender distribution. Nord-Trøndelag County is considered to be fairly representative of Norway in most respects, with the exceptions of the lack of major cities, and a relatively small immigrant population. This allowed us to explore the dimensional structure of the items in each of the main occupational groups (ISCO 0–9). However, age restriction is a limiting factor. The age group 30–69 includes most of the occupationally active, however, the youngest part of the working population is not represented in the study. In addition, the mean age of the respondents is higher than in most other studies [12–14]. However, research indicates that *demand*, *support* and *control* scores are not highly age dependent [50,51].

Despite the broad inclusion criteria in HUNT4, one must consider the issue of selection bias seen in population-based cohort studies. Participation in such studies is influenced by factors such as socioeconomic status, health status, age and sex [52]. Non-participation in HUNT has been associated with a lower socioeconomic status, a higher prevalence of several chronic diseases, and higher mortality [18,53]. It is important to be aware of this selection bias and appreciate the effect it may have on the representativeness of the data.

Even though the data was not MCAR, listwise deletion of participants with one or more missing values was considered most appropriate. Imputation could generate unreliable results because only participants who were “working with others” were asked to respond to the support items. Consequently, the percentage of missing cases was higher on the support items, and closer inspection of the trend in missing values demonstrated a relationship between missing values and occupational group. The highest proportion of missing responses to the support items was amongst workers in ISCO group 6, which include skilled agricultural, forestry and fishery workers. These are jobs that often involve a lot of solitary work [54], which could have made it challenging for these study participants to respond to the items regarding collegiality and support at work. The link between the missing data and the occupations should be considered when using these items to assess psychosocial work environment as some occupational groups may find it difficult to respond to the support items because they work alone. Listwise deletion could introduce bias when the data is not missing completely at random [55], however, because we had less than 10% missing responses it was considered not to significantly influence the results [56]. In addition, the CFA analyses showed that despite the high number of missing values in occupational group 6, the items still had the same dimensional structure as in the other occupational groups and in the sample as a whole. This indicates that listwise deletion of the missing values did not influence the dimensional structure of the DCSQ items used in HUNT4. Other methods of handling missing data, such as pairwise deletion or imputation, may be considered in future studies using the DCSQ items to reduce bias listwise deletion may cause. However, the method chosen will depend on how the items are used, and the population studies. In addition, the underlying criteria set for responding to the support items should be considered when imputing missing values, to ensure the imputation does not generate unreliable results.

## Conclusion

The results indicate that the items from the Swedish DCSQ used in HUNT4 can be used to assess the aspects of demand, control and social support at work in an occupationally heterogenous and homogeneous sample. Eight of the nine items show the same three-dimensional structure as described in previous research done on the full 17 items questionnaire. The poor fit of the item “Work creativity” in the three-factor model could support the theory of a four-factor model as this item belongs to a different subcategory of the control factor than the other two items in the dimension.

## Supporting information

S1 Table Exploratory factor analysis of the nine demand-control-support items used in The Trøndelag Health study in 2017–2019 (HUNT4). (DOCX)

S2 Table Confirmatory factor analysis of the Swedish Demand-Control-Support Questionnaire items used in the Trøndelag Health Study in 2017–2019 (HUNT4) on ISCO groups 0–4. (DOCX)

S3 Table Confirmatory factor analysis of the Swedish Demand-Control-Support Questionnaire items used in the Trøndelag Health Study in 2017–2019 (HUNT4) on ISCO groups 5–9. (DOCX)

## References

[1] Michie S. Causes and management of stress at work. Occup Environ Med. 2002;59(1):67–72. doi: 10.1136/oem.59.1.67 11836475 PMC1740194

[2] Häusser JA, Mojzisch A, Niesel M, Schulz-Hardt S. Ten years on: A review of recent research on the job demand-control (-support) model and psychological well-being. Work Stress. 2010;24(1):1–35. doi: 10.1080/02678371003683747

[3] Karasek RA. Job demands, job decision latitude, and mental strain: Implications for job redesign. Admin Sci Quart 1979;24(2):285–308. doi: 10.2307/2392498

[4] Karasek R, Theorell T. Healthy work: stress, productivity, and the reconstruction of working life. New York: Basic Books; 1990.

[5] Johnson JV, Hall EM. Job strain, work place social support, and cardiovascular disease: a cross-sectional study of a random sample of the Swedish working population. Am J Public Health. 1988;78(10):1336–42. doi: 10.2105/ajph.78.10.1336 3421392 PMC1349434

[6] Sanne B, Torp S, Mykletun A, Dahl AA. The Swedish Demand-Control-Support Questionnaire (DCSQ): factor structure, item analyses, and internal consistency in a large population. Scand J Public Health. 2005;33(3):166–74. doi: 10.1080/14034940410019217 16040456

[7] Van der Doef M, Maes S. The job demand-control (-support) model and psychological well-being: a review of 20 years of empirical research. Work Stress. 1999;13(2):87–114. doi: 10.1080/026783799296084

[8] Berkman LF, Kawachi I, Theorell T. Working conditions and health In: Berkman LF, Kawachi I, Glymour M, editors. Social epidemiology 2nd ed: Oxford University Press 2014. p. 153–81.

[9] Theorell T, Perski A, Åkerstedt T, Sigala F, Ahlberg-Hulten G, Svensson J, Eneroth P. Changes in job strain in relation to changes in physiological state: A longitudinal study. Scand J Work Env Hea. Jun 1988;14(3):189–96. doi: 10.5271/sjweh.1932 3393855

[10] Theorell T. The demand-control-support model for studying health in relation to the work environment: an interactive model. In: Orth-Gomer K, Schneiderman N, editors. Behavioral medicine approaches to cardiovascular disease prevention. New York, USA: Psychology Press 1996. p. 69–85.

[11] Chungkham HS, Ingre M, Karasek R, Westerlund H, Theorell T. Factor structure and longitudinal measurement invariance of the demand control support model: An evidence from the Swedish longitudinal occupational survey of health (SLOSH). Plos One. 2013;8(8):e70541–e. doi: 10.1371/journal.pone.0070541 23950957 PMC3741382

[12] Hökerberg YHM, Aguiar OB, Reichenheim M, Faerstein E, Valente JG, Fonseca MdJ, Passos SRL. Dimensional structure of the demand control support questionnaire: a Brazilian context. Int Arch Occ Env Hea. 2010;83(4):407–16. doi: 10.1007/s00420-009-0488-4 19941002

[13] Mauss D, Herr RM, Theorell T, Angerer P, Li J. Validating the Demand Control Support Questionnaire among white-collar employees in Switzerland and the United States. J Occup Med Toxicol. 2018;13(7). doi: 10.1186/s12995-018-0188-7 29449870 PMC5812053

[14] Alfaro-Díaz C, Esandi N, Pueyo-Garrigues M, Pardavila-Belio MI, Canga-Armayor N, Canga-Armayor A. Translation and psychometric validation of the Spanish version of the Demand–Control–Support Questionnaire (DCSQ) for nursing professionals. J Nurs Manage. 2021;29:1130–40. doi: 10.1111/jonm.13251 33438261

[15] Demerouti E, Nachreiner F, Schaufeli W. The Job Demands–Resources model of burnout. J Appl Psychol. 2001;86(3):499–512. doi: 10.1037/0021-9010.86.3.499 11419809

[16] Taris TW. Chapter 4: Models in work and health research: the JDC(S), ERI and JD-R frameworks. In: Burke RJ, Page KM, editors. Research Handbook on Work and Well-Being. Cheltenham, UK: Edward Elgar Publishing; 2017.

[17] Sanne B, Mykletun A, Dahl AA, Moen BE, Tell GS. Testing the Job Demand-Control-Support model with anxiety and depression as outcomes: the Hordaland Health Study. Occup Med. 2005;55(6):463–73. doi: 10.1093/occmed/kqi071 15845554

[18] Åsvold BO, Langhammer A, Rehn TA, Kjelvik G, Grøntvedt TV, Sørgjerd EP, et al. Cohort profile update: The HUNT Study, Norway. Int J Epidemiol. 2023;52(1):e80–e91. doi: 10.1093/ije/dyac095 . 35578897 PMC9908054

[19] The HUNT Research Centre. The HUNT databank: Swedish demand-control-support questionnaire [cited 2024 June 8]. https://hunt-db.medisin.ntnu.no/hunt-db/instrument/DCSQ.

[20] Strømholm T, Pape K, Ose S, Krokstad S, Bjørngaard J. Psychosocial working conditions and sickness absence in a general population: A cohort study of 21,834 workers in Norway (The HUNT Study). J Occup Environ Med. 2015;57(4):386–92. doi: 10.1097/JOM.0000000000000362 25851186

[21] The HUNT Research Centre. The Trøndelag Health Study Surveys [cited 2024 24 June]. https://www.ntnu.edu/web/hunt/hunt-surveys.

[22] Krokstad S, Sund ER, Kvaløy K, Rangul V, Næss M. HUNT for better public health. Scand J Public Healt. 2022;50(7):968–71. doi: 10.1177/14034948221102309 . 36113104 PMC9578099

[23] Statistics Norway. Standard for yrkesklassifisering [Standard Classification of Occupations]: Statistics Norway; 1998 [cited 2023 Sep 8]. https://www.ssb.no/klass/klassifikasjoner/7/versjon/34.

[24] R Core Team. R: a language and environment for statistical computing. Vienna, Austria; 2024. www.R-project.org.

[25] Rosseel Y. lavaan: An R package for structural equation modeling. J Stat Softw. 2012;48:1–36. doi: 10.18637/jss.v048.i02

[26] Jorgensen TD, Pornprasertmanit S, Schoemann AM, Rosseel Y. semTools: Useful tools for structural equiation modeling. 2022 [cited 2023 8 Sep]. https://CRAN.R-project.org/package=semTools.

[27] Bliese P. multilevel: Multilevel Functions 2022 [cited 2023 8 Sep]. https://CRAN.R-project.org/package=multilevel.

[28] Revelle W. psych: procedures for psychological, psychometric, and personality research Evanston, Illinois Northwestern University 2024 [cited 2024 1 Mar]. https://CRAN.R-project.org/package=psych.

[29] Lishinski A. lavaanPlot: path diagrams for "Lavaan" models via "DiagrammeR" 2024 [cited 2024 1 Mar]. https://github.com/alishinski/lavaanPlot.

[30] Little RJA. A test of missing completely at random for multivariate data with missing values. J Am Stat Assoc. 1988;83(404):1198–202. doi: 10.1080/01621459.1988.10478722

[31] Tierney N, Cook D. Expanding tidy data principles to facilitate missing data exploration, visualization and assessment of imputations. J Stat Softw. 2023;105(7):1–31. doi: 10.18637/jss.v105.i0736798141

[32] Hair J, Black W, Babin B, Anderson R, Tatham R. SEM: confirmatory factor analysis. In: Hair J, Black W, Babin B, Anderson R, Tatham R, editors. Multivariate data analysis. 6th ed. Upper Saddle River, N.J.: Prentice Hall; 2006. p. 770–842.

[33] Holgado–Tello FP, Chacón–Moscoso S, Barbero–García I, Vila–Abad E. Polychoric versus Pearson correlations in exploratory and confirmatory factor analysis of ordinal variables. Qual Quant. 2010;44(1):153–66. doi: 10.1007/s11135-008-9190-y

[34] Tabachnick BG, Fidell LS. Principal components and factor analysis. In: Tabachnick BG, Fidell LS, editors. Using multivariate statistics 6th ed. Boston, USA: Pearson; 2013. p. 612–81.

[35] Li CH. Confirmatory factor analysis with ordinal data: Comparing robust maximum likelihood and diagonally weighted least squares. Behav Res Methods. 2016;48(3):936–49. doi: 10.3758/s13428-015-0619-7 26174714

[36] Lt Hu, Bentler PM. Cutoff criteria for fit indexes in covariance structure analysis: Conventional criteria versus new alternatives. Struct Equ Modeling. 1999;6(1):1–55. doi: 10.1080/10705519909540118

[37] Field AP. Discovering statistics using SPSS. 2nd ed. London, England: Sage Publications; 2005.

[38] The HUNT Research Centre. HUNT4 [cited 2023 28. August]. https://www.ntnu.no/hunt/hunt4.

[39] Lov om medisinsk og helsefaglig forskning [The health research act]. LOV-2008-06-20-44 [cited 2023 24 Jul]. https://lovdata.no/dokument/NL/lov/2008-06-20-44.

[40] Kost RG, de Rosa JC. Impact of survey length and compensation on validity, reliability, and sample characteristics for ultrashort-, short-, and long-research participant perception surveys. J Clin Transl Sci. 2018;2(1):31–7. doi: 10.1017/cts.2018.18 30393572 PMC6208327

[41] Matthews LM, Rutherford BN. The impact of skill discretion and work demands on salesperson job satisfaction: the mediating influence of the burnout facets. J Pers Sell Sales Manag. 2020;41(1):17–27. doi: 10.1080/08853134.2020.1815542

[42] Viotti S, Converso D. Relationship between job demands and psychological outcomes among nurses: does skill discretion matter? Int J Occup Med Env. 2016;29(3):439–60. doi: 10.13075/ijomeh.1896.00520 26988883

[43] Ganster DC, Fusilier MR. Control in the workplace. In: Cooper C, editor. International review of industrial and organizational psychology 1989. Oxford, England: John Wiley & Sons; 1989. p. 235–80.

[44] Oven A, Domajnko B. Job satisfaction and creativity at work among occupational therapy practitioners: A mixed-methods study. Work. 2021;69(4):1351–62. doi: 10.3233/WOR-213555 . 34421002

[45] Theorell T, Karasek RA. Current issues relating to psychosocial job strain and cardiovascular disease research. J Occup Health Psych. 1996;1(1):9–26. doi: 10.1037//1076-8998.1.1.9 9547038

[46] Marsh HW, Hau K-T, Balla JR, Grayson D. Is more ever too much? The number of indicators per factor in confirmatory factor analysis. Multivariate Behav Res. 1998;33(2):181–220. doi: 10.1207/s15327906mbr3302_1 26771883

[47] Koran J. Indicators per factor in confirmatory factor analysis: More is not always better. Struct Equ Modeling. 2020;27(5):765–72. doi: 10.1080/10705511.2019.1706527

[48] Hämmig O. Health and well-being at work: The key role of supervisor support. SSM Popul Health. 2017;3:393–402. doi: 10.1016/j.ssmph.2017.04.002 29349232 PMC5769068

[49] Inoue R, Hikichi H, Inoue A, Kachi Y, Eguchi H, Watanabe K, et al. Workplace Social Support and Reduced Psychological Distress: A 1-Year Occupational Cohort Study. J Occup Environ Med. 2022;64(11):700–4. doi: 10.1097/JOM.0000000000002675 35959920

[50] Karasek R, Brisson C, Kawakami N, Houtman I, Bongers P, Amick B. The Job Content Questionnaire (JCQ): an instrument for internationally comparative assessments of psychosocial job characteristics. J Occup Health Psychol. 1998;3(4):322–55. doi: 10.1037//1076-8998.3.4.322 9805280

[51] Pelfrene E, Vlerick P, Mak R, Smet P, Kornitzer M, De Backer G. Scale reliability and validity of the Karasek ’Job Demand-Control-Support’ model in the Belstress study. Work Stress. 2001;15(4):297–313. doi: 10.1080/02678370110086399

[52] Enzenbach C, Wicklein B, Wirkner K, Loeffler M. Evaluating selection bias in a population-based cohort study with low baseline participation: the LIFE-Adult-Study. BMC Med Res Methodol. 2019;19:135. doi: 10.1186/s12874-019-0779-8 31262266 PMC6604357

[53] Langhammer A, Krokstad S, Romundstad P, Heggland J, Holmen J. The HUNT study: participation is associated with survival and depends on socioeconomic status, diseases and symptoms. BMC Med Res Methodol. 2012;12. Epub 20120914. doi: 10.1186/1471-2288-12-143 . 22978749 PMC3512497

[54] Arbeidsmiljøportalen. Fakta om arbeidsmiljøet i fiske, akvakultur, jord- og skogbruk [cited 2023 05. Sept.]. https://www.arbeidsmiljoportalen.no/bransje/landbruk/fakta-om-bransjen.

[55] Kline RB. Data Preparation. In: Kline RB, editor. Principles and practice of structural equation modeling. 5th ed. New York: Guilford publications; 2023. p. 46–66.

[56] Bennett DA. How can I deal with missing data in my study? Aust N Z J Public Health 2001;25(5):464–9. doi: 10.1111/j.1467-842X.2001.tb00294.x 11688629

