# Supplementary material for: Dimensional structure of the items from The Swedish Demand-Control-Support Questionnaire (DCSQ) used in The HUNT Study
Source: PLoS One. 2024 Sep 26;19(9):e0308611. doi: 10.1371/journal.pone.0308611 (PMC11426464; doi:10.1371/journal.pone.0308611)
Supplement: S1 Table — (DOCX) [file pone.0308611.s001.docx]

**S1 Table. Exploratory factor analysis of the nine demand-control-support items used in HUNT4 (2017–2019).**

|  | Items | F1 | F2 | F3 |
| --- | --- | --- | --- | --- |
| Demand |  |  |  |  |
|  | *Work Fast* |  | 0.788 |  |
|  | *Work Hard* |  | 0.752 |  |
|  | *Work High* |  | 0.648 |  |
| Control |  |  |  |  |
|  | *Work creativity* |  |  | 0.448 |
|  | *Work how* |  |  | 0.893 |
|  | *Work what* |  |  | 0.861 |
| Support |  |  |  |  |
|  | *Work collegiality* | 0.872 |  |  |
|  | *Work support* | 0.890 |  |  |
|  | *Work welfare* | 0.946 |  |  |
| Variance explained (%) |  | 27.2 | 19.5 | 19.0 |

Exploratory factor analysis (EFA) using principal component analysis with oblique rotation. Only factor loadings >0.4 are included in the table.
