# Supplementary material for: Dimensional structure of the items from The Swedish Demand-Control-Support Questionnaire (DCSQ) used in The HUNT Study
Source: PLoS One. 2024 Sep 26;19(9):e0308611. doi: 10.1371/journal.pone.0308611 (PMC11426464; doi:10.1371/journal.pone.0308611)
Supplement: S2 Table — (DOCX) [file pone.0308611.s002.docx]

|  | **ISCO 0** | | | | **ISCO 1** | | | | **ISCO 2** | | | | **ISCO 3** | | | | **ISCO 4** | | | |
| --- | --- | --- | --- | --- | --- | --- | --- | --- | --- | --- | --- | --- | --- | --- | --- | --- | --- | --- | --- | --- |
|  | Model 1 | | Model 2 | | Model 1 | | Model 2 | | Model 1 | | Model 2 | | Model 1 | | Model 2 | | Model 1 | | Model 2 | |
|  | **λ** | **δ** | **λ** | **δ** | **λ** | **δ** | **λ** | **δ** | **λ** | **δ** | **λ** | **δ** | **λ** | **δ** | **λ** | **δ** | **λ** | **δ** | **λ** | **δ** |
| **Demand** |  | | | | | | | | | | | | | | | | | | | |
| Work fast | 0.838 | 0.298 | 0.843 | 0.290 | 0.824 | 0.321 | 0.835 | 0.303 | 0.797 | 0.364 | 0.799 | 0.362 | 0.800 | 0.360 | 0.808 | 0.347 | 0.821 | 0.326 | 0.827 | 0.316 |
| Work hard | 0.864 | 0.253 | 0.861 | 0.259 | 0.735 | 0.460 | 0.728 | 0.470 | 0.721 | 0.481 | 0.719 | 0.484 | 0.707 | 0.501 | 0.701 | 0.509 | 0.735 | 0.460 | 0.731 | 0.465 |
| Work effort | 0.732 | 0.465 | 0.731 | 0.466 | 0.550 | 0.698 | 0.544 | 0.704 | 0.598 | 0.643 | 0.599 | 0.642 | 0.635 | 0.596 | 0.633 | 0.599 | 0.709 | 0.497 | 0.707 | 0.500 |
| **Control** |  | | | | | | | | | | | | | | | | | | | |
| Work creativity | 0.373 | 0.861 | - | - | 0.309 | 0.905 | - | - | 0.352 | 0.876 | - | - | 0.376 | 0.859 | - | - | 0.322 | 0.896 | - | - |
| Work how | 0.911 | 0.169 | 0.840 | 0.294 | 0.941 | 0.115 | 1.043 | -0.087 | 0.988 | 0.025 | 0.994 | 0.012 | 0.944 | 0.108 | 0.932 | 0.131 | 0.936 | 0.124 | 1.131 | -0.278 |
| Work what | 0.858 | 0.264 | 0.922 | 0.149 | 0.825 | 0.320 | 0.744 | 0.446 | 0.805 | 0.352 | 0.792 | 0.373 | 0.819 | 0.328 | 0.825 | 0.320 | 0.817 | 0.332 | 0.672 | 0.548 |
| **Support** |  | | | | | | | | | | | | | | | | | | | |
| Work collegiality | 0.887 | 0.213 | 0.888 | 0.212 | 0.881 | 0.223 | 0.881 | 0.224 | 0.879 | 0.228 | 0.879 | 0.228 | 0.888 | 0.212 | 0.888 | 0.212 | 0.898 | 0.193 | 0.898 | 0.193 |
| Work support | 0.920 | 0.154 | 0.919 | 0.155 | 0.901 | 0.189 | 0.901 | 0.188 | 0.893 | 0.202 | 0.893 | 0.202 | 0.903 | 0.185 | 0.903 | 0.185 | 0.904 | 0.183 | 0.904 | 0.183 |
| Work welfare | 0.915 | 0.162 | 0.915 | 0.162 | 0.953 | 0.091 | 0.953 | 0.092 | 0.945 | 0.107 | 0.945 | 0.107 | 0.954 | 0.090 | 0.954 | 0.090 | 0.939 | 0.118 | 0.939 | 0.118 |
| **Goodness of fit indices** | | | | | | | | | | | | | | | | | | | | |
| TLI | 0.967 | | 1.001 | | 0.969 | | 0.999 | | 0.987 | | 0.998 | | 0.985 | | 0.998 | | 0.979 | | 1.000 | |
| CFI | 0.978 | | 1.000 | | 0.979 | | 1.000 | | 0.991 | | 0.999 | | 0.990 | | 0.999 | | 0.986 | | 1.000 | |
| RMSEA (90% CI) | 0.122 (0.098–0.147) | | 0.000 (0.000–0.053) | | 0.094 (0.086–0.102) | | 0.015 (0.000–0.028) | | 0.069 (0.063–0.075) | | 0.029 (0.022–0.037) | | 0.078 (0.074–0.083) | | 0.029 (0.023–0.034) | | 0.099 (0.089–0.110) | | 0.006 (0.000–0.029) | |
| SRMR | 0.098 | | 0.046 | | 0.081 | | 0.025 | | 0.059 | | 0.028 | | 0.065 | | 0.025 | | 0.078 | | 0.025 | |

**S2 Table.** **Confirmatory factor analysis of the Swedish Demand-Control-Support Questionnaire items used in the Trøndelag Health Study in 2017–2019 (HUNT4) on ISCO groups 0-4.**

Confirmatory factor analysis with standardised factor loadings (λ), standardised factor correlations and standardised residual variance (δ). Model 1 included the item “Work creativity”, this item was removed from Model 2. TLI = Tucker Lewis index. CFI = Comparative fit index. RMSEA = Root mean square error of approximation. SRMR = Standardised root mean squared residual. ISCO = International standard classification of occupations.
