# Supplementary material for: Dimensional structure of the items from The Swedish Demand-Control-Support Questionnaire (DCSQ) used in The HUNT Study
Source: PLoS One. 2024 Sep 26;19(9):e0308611. doi: 10.1371/journal.pone.0308611 (PMC11426464; doi:10.1371/journal.pone.0308611)
Supplement: S3 Table — (DOCX) [file pone.0308611.s003.docx]

**S3 Table.** **Confirmatory factor analysis of the Swedish Demand-Control-Support Questionnaire items used in the Trøndelag Health Study in 2017–2019 (HUNT4) on ISCO groups 5-9.**

|  | **ISCO 5** | | | | **ISCO 6** | | | | **ISCO 7** | | | | **ISCO 8** | | | | **ISCO 9** | | | |
| --- | --- | --- | --- | --- | --- | --- | --- | --- | --- | --- | --- | --- | --- | --- | --- | --- | --- | --- | --- | --- |
|  | **Model 1** | | **Model 2** | | **Model 1** | | **Model 2** | | **Model 1** | | **Model 2** | | **Model 1** | | **Model 2** | | **Model 1** | | **Model 2** | |
|  | **λ** | **δ** | **λ** | **δ** | **λ** | **δ** | **λ** | **δ** | **λ** | **δ** | **λ** | **δ** | **λ** | **δ** | **λ** | **δ** | **λ** | **δ** | **λ** | **δ** |
| **Demand** |  | | | | | | | | | | | | | | | | | | | |
| Work fast | 0.834 | 0.304 | 0.840 | 0.295 | 0.753 | 0.432 | 0.759 | 0.423 | 0.810 | 0.344 | 0.808 | 0.348 | 0.776 | 0.398 | 0.792 | 0.373 | 0.723 | 0.478 | 0.727 | 0.471 |
| Work hard | 0.820 | 0.328 | 0.814 | 0.337 | 0.852 | 0.273 | 0.857 | 0.265 | 0.820 | 0.328 | 0.821 | 0.326 | 0.896 | 0.198 | 0.881 | 0.224 | 0.916 | 0.160 | 0.911 | 0.169 |
| Work effort | 0.629 | 0.604 | 0.630 | 0.604 | 0.682 | 0.535 | 0.668 | 0.553 | 0.661 | 0.563 | 0.663 | 0.561 | 0.593 | 0.648 | 0.589 | 0.653 | 0.626 | 0.608 | 0.626 | 0.608 |
| **Control** |  | | | | | | | | | | | | | | | | | | | |
| Work creativity | 0.342 | 0.883 | - | - | 0.494 | 0.756 | - | - | 0.450 | 0.797 | - | - | 0.567 | 0.678 | - | - | 0.203 | 0.959 | - | - |
| Work how | 0.881 | 0.223 | 0.873 | 0.238 | 0.926 | 0.143 | 0.962 | 0.074 | 0.921 | 0.152 | 0.855 | 0.268 | 0.867 | 0.248 | 0.838 | 0.297 | 0.880 | 0.226 | 0.956 | 0.086 |
| Work what | 0.849 | 0.280 | 0.850 | 0.277 | 0.799 | 0.362 | 0.801 | 0.359 | 0.805 | 0.353 | 0.860 | 0.261 | 0.858 | 0.263 | 0.904 | 0.183 | 0.841 | 0.293 | 0.772 | 0.404 |
| **Support** |  | | | | | | | | | | | | | | | | | | | |
| Work collegiality | 0.886 | 0.215 | 0.886 | 0.215 | 0.855 | 0.269 | 0.855 | 0.270 | 0.853 | 0.272 | 0.854 | 0.271 | 0.862 | 0.256 | 0.863 | 0.256 | 0.893 | 0.202 | 0.893 | 0.202 |
| Work support | 0.894 | 0.200 | 0.894 | 0.201 | 0.856 | 0.267 | 0.857 | 0.266 | 0.863 | 0.255 | 0.863 | 0.255 | 0.856 | 0.267 | 0.856 | 0.267 | 0.932 | 0.132 | 0.932 | 0.132 |
| Work welfare | 0.941 | 0.115 | 0.941 | 0.115 | 0.922 | 0.150 | 0.921 | 0.151 | 0.932 | 0.132 | 0.932 | 0.132 | 0.957 | 0.084 | 0.957 | 0.085 | 0.996 | 0.007 | 0.996 | 0.007 |
| **Goodness of fit indices** | | | | | | | | | | | | | | | | | | | | |
| TLI | 0.986 | | 0.998 | | 0.971 | | 0.998 | | 0.981 | | 0.999 | | 0.983 | | 0.998 | | 0.990 | | 1.000 | |
| CFI | 0.991 | | 0.999 | | 0.981 | | 0.999 | | 0.988 | | 0.999 | | 0.989 | | 0.999 | | 0.994 | | 1.000 | |
| RMSEA (90% CI) | 0.074 (0.069–0.079) | | 0.028 (0.022–0.035) | | 0.096 (0.085–0.107) | | 0.031 (0.014–0.046) | | 0.071 (0.063–0.079) | | 0.017 (0.000–0.029) | | 0.080 (0.071–0.090) | | 0.027 (0.013–0.041) | | 0.080 (0.067–0.093) | | 0.000 (0.000–0.026) | |
| SRMR | 0.061 | | 0.025 | | 0.083 | | 0.039 | | 0.061 | | 0.024 | | 0.063 | | 0.030 | | 0.068 | | 0.026 | |

Confirmatory factor analysis with standardised factor loadings (λ), standardised factor correlations and standardised residual variance (δ). Model 1 included the item “Work creativity”, this item was removed from Model 2. TLI = Tucker Lewis index. CFI = Comparative fit index. RMSEA = Root mean square error of approximation. SRMR = Standardised root mean squared residual. ISCO = International standard classification of occupations.
